# Successful radiofrequency catheter ablation of Wolff-Parkinson-White syndrome in a patient with dextrocardia: A case report

**DOI:** 10.3389/fcvm.2022.1006435

**Published:** 2022-10-24

**Authors:** Lei Zhao, Ruibin Li, Long Bai, Dong Wang, Jidong Zhang, Xiaohong Yang

**Affiliations:** Department of Cardiology, The Second Hospital of Hebei Medical University, Shijiazhuang, China

**Keywords:** dextrocardia, Wolff-Parkinson-White (WPW) syndrome, radiofrequency catheter ablation, tachycardia, congenital heart malformation

## Abstract

**Background:**

Dextrocardia is a congenital heart malformation with a low incidence that occurs in only 1 in 10,000–12,000 people. Wolff-Parkinson-White (WPW) syndrome is a congenital condition with additional accessory pathways between the atria and the ventricle, which affects up to three in 1,000 people worldwide. Experience of radiofrequency catheter ablation in patients with WPW syndrome and dextrocardia is scarce due to its rare incidence.

**Case presentation:**

A 39-year-old female was hospitalized due to two episodes of palpitations in the latest 2 months. The morphology of the P-QRS-T complex of lead aVR and aVL, II, and III were presented invertedly as common conditions, and shortened P-R interval and a characteristic “delta” wave were shown on the electrocardiogram (EGM). The patient with dextrocardia and situs invertus malposition was confirmed by chest-X ray, cardiac color Doppler echocardiography. The patient was diagnosed with WPW syndrome with dextrocardia and underwent radiofrequency catheter ablation (RFCA) successfully. In this case, the key to the success of RFCA is to understand the anatomical structure of the heart and the great vessels before the operation and make a personalized operative plan.

**Conclusion:**

Catheter ablation for tachycardia patients with dextrocardia is efficient and safe. For patients with dextrocardia, the key to successful ablation was adjusting for projection angulation and different catheter manipulation compared with a standard case because of the mirror image of a normal heart.

## Introduction

Dextrocardia with situs inversus is a rare congenital malformation characterized by the mirror-image location of the heart and viscera. Additionally, Wolff-Parkinson-White (WPW) is a congenital abnormality caused by an accessory pathway between the atria and ventricles that bypasses the atrioventricular (AV) node and the His-Purkinje system. WPW exists in 0.1–0.3% of the population (1, 2). Therefore, the morbidity rate of the two conditions is very low. Due to its rare incidence, the experience of radiofrequency catheter ablation (RFCA) of tachycardia in patients with dextrocardia is limited. Here, we report a WPW syndrome case in a patient with dextrocardia who successfully underwent RFCA.

## Case presentation

**FIGURE 1:**
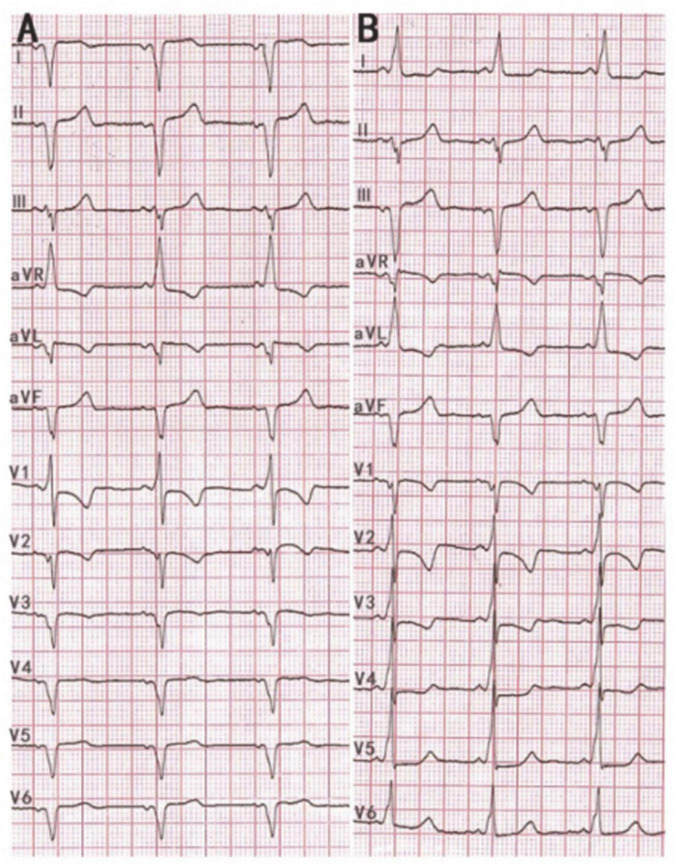
**(A)** 12 lead ECG with standard lead placement showing sinus rhythm with a heart rate of 68 beats per minute. ECG revealed inverted P-QRS-T complex waves in lead I and inverted P-QRS in lead aVL. Notably, the P-QRS-T complex in lead aVR and aVL appear reversed compared to their usual relationship. Likewise, the morphology of the P-QRS-T complex of lead II and III was also invertedly compared to their usual relationship. The ECG shows an RS morphology in V1, a reversed R wave from V2 to V6 leads, a shortened P-R interval, and a characteristic “delta” wave. **(B)** 12 lead ECG with reverse lead placement and right precordial leads V3R-V6R. The ECG showed a positive P-QRS complex in lead I. The morphology of the P-QRS-T complex of lead aVR and aVL, lead II, and III were reversed compared to their morphologies in panel **(A)**. R wave amplitude increases gradually, and the S wave decreases gradually in the precordial leads and the “delta” wave in 12 leads.

An Asian 39-year-old female was admitted to the Second Hospital of Hebei Medical University in January 2022 due to two episodes of palpitations in the past 2 months. During the physical examination on admission, her resting heart rate was 71 beats per minute, her blood pressure was 117/64 mm Hg, and an apex beat was identified on the right side of the chest with no murmur. We completed the 12-lead electrocardiogram, chest-X ray, echocardiography, and blood examinations to evaluate the patient’s condition. A twelve-lead EGM with standard lead placement showed inverted P-QRS-T complex waves in lead I and an inverted P-QRS in lead aVL. Notably, the P-QRS-T complex in lead aVR and aVL appear reversed compared to their usual relationship. Likewise, the morphology of the P-QRS-T complex of lead II and III were also reversed. The EGM shows an RS morphology in V1, a reversed R wave from V2 to V6, a shortened P-R interval, and a characteristic “delta” wave (a wide and initially slurred QRS complex) in precordial leads (Figure 1A). We completed a 12-lead EGM with reversed lead placement and right precordial leads V3R-V6R. From our results, the EGM showed a positive P-QRS complex in lead I. The morphology of the P-QRS-T complex of lead aVR and aVL, lead II, and III were reversed, as presented in Figure 1A. Furthermore, the R wave amplitude increased gradually, and the S wave decreased gradually in the precordial leads (Figure 1B).

**FIGURE 2:**
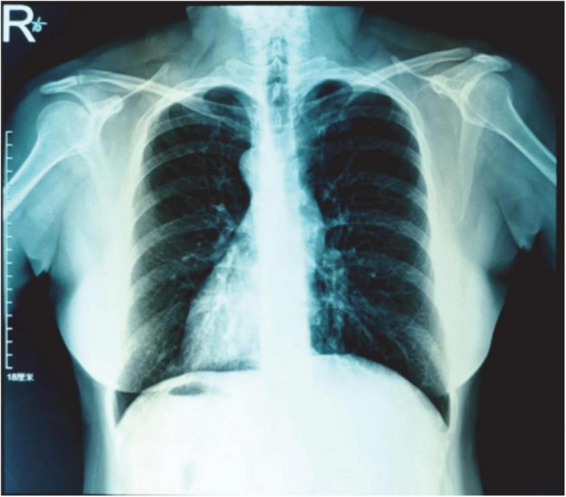
The PA view chest X-ray shows shadows of the heart and aortic arch in the right hemithorax along with gas in the fundus of the stomach on the right side.

The posteroanterior (PA) view chest X-ray shows shadows of the heart and aortic arch in the right hemithorax along with gas in the fundus of the stomach on the right side (Figure 2).

Echocardiography showed a normal size of each heart cavity and normal biventricular functions with an ejection fraction of 76.2%. No regional wall motion abnormalities and septal defects were detected. Furthermore, laboratory examinations showed no abnormalities.

Based on the above clinical manifestations and examinations, we diagnosed the patient with Wolff–Parkinson–White syndrome combined with dextrocardia. The following operation strategy of RFCA was performed successfully under local anesthesia.

## Electrophysiological study and radiofrequency catheter ablation

**FIGURE 3:**
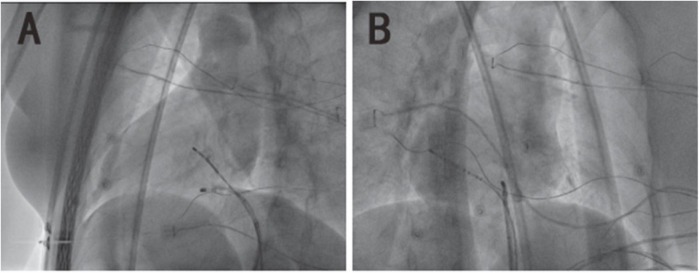
The X-ray image of mirror dextrocardia in the left anterior oblique view of 45° and right-anterior oblique view of 35°. **(A)** The image of dextrocardia in the left anterior oblique is presented as a common heart in the regular right anterior oblique. **(B)** The image of dextrocardia in the right anterior oblique is presented as a common heart in the regular left anterior oblique.

**FIGURE 4:**
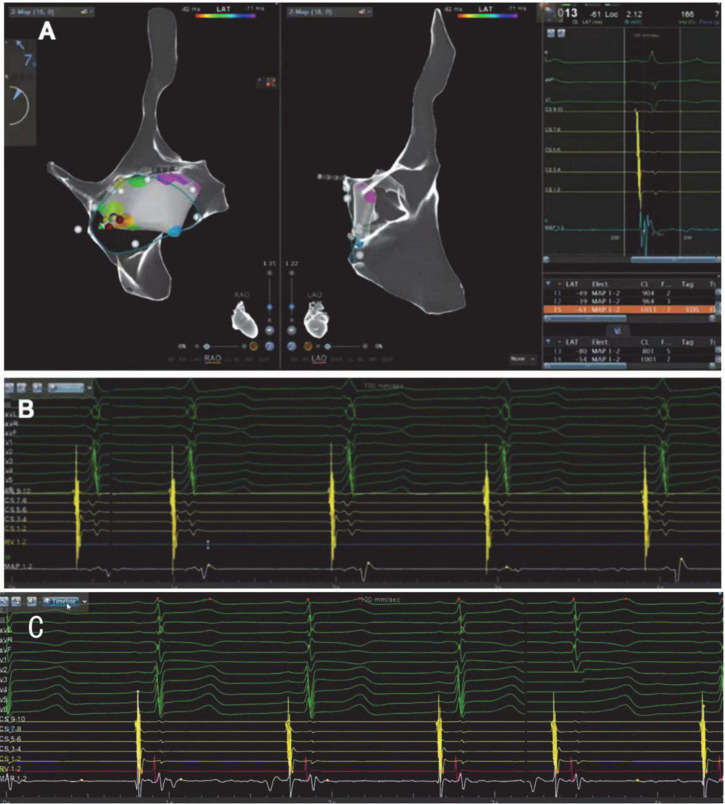
**(A)** CARTO electroanatomical 3-dimensional(3D) mapping system showing the right atrium is a mirror image of a normal heart in the right-anterior oblique view(RAO)and the left-anterior oblique view (LAO). **(B)** ECG (before RFCA) with reverse lead placement and right precordial leads V3R-V6R shows an inferoseptal tricuspid annulus accessory pathway. The prominent early V wave in 7/8 bipolar potentials of 10 polar coronary sinus electrodes also indicates the accessory pathway was in the inferoseptal nearby the coronary sinus ostium. Ventricular activation was marked by the red arrow. **(C)** The ECG after the radiofrequency catheter ablation shows “delta” wave disappeared, indicating that the procedure was successful.

This procedure was performed under standard local anesthesia and guided by CARTO electroanatomical three-dimensional (3D) mapping system. A steerable Decapolar catheter (XTTM, Bard Electrophysiology, Lowell, MA, USA) and 4 polar catheters (AVAIL, Electrophysiology Cather, Biosense Webster, USA) were positioned in the coronary sinus (CS) through the right femoral vein. An 8.5-Fr long sheath (SL1, St. Jude Medical, MN, USA) was advanced into the right atrium through the right femoral vein, and a Thermocool Smart touch catheter was introduced to the region of His-bundle from SL1 (Figure 3). Supraventricular tachycardia was induced with the frequency of 180 beats per minute by ventricular S1S1, and A was reset by right ventricular apex entrainments and RS2 stimuli. The patient was diagnosed with dextrocardia with a posteroseptal tricuspid annulus accessory pathway by the intracardiac EGM (Figures 4A,B). After ablation at 43°C and 30W for 5 s, the accessory pathway was blocked and continued to ablate for 240 s. Retrograde Wenckebach conduction was stimulated by ventricular S1S1 for 400 ms, decreased conduction was stimulated by coronary sinus S1S1 stimulation, and Wenckebach conduction was found for 300 ms. No ventricular conduction with atria stimulated by S1S2 of 450/300 ms, indicating that the procedure was successful. Delta wave disappeared on intracardiac and surface EGM (Figure 4C).

**FIGURE 5:**
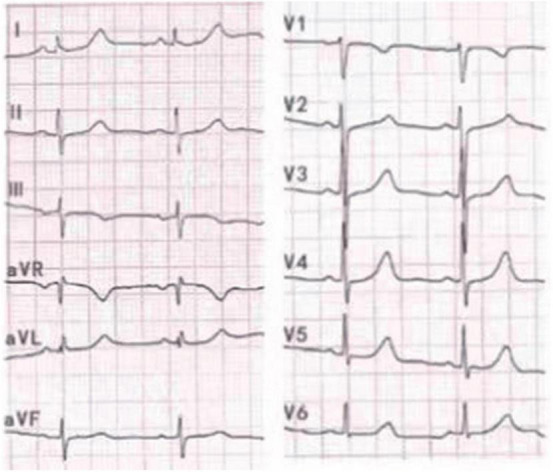
No characteristic “delta” wave was seen, and normal R/S transition was shown in precordial leads in 12 lead ECG with reverse lead placement.

After a 6-month of follow-up, the patient did not have complaints of onset palpitations, and no arrhythmia was observed on the EGM (Figure 5).

## Discussion

Dextrocardia is a rare congenital abnormality that occurs in 1 in 10,000–12,000 people (3). There are three main types of dextrocardia. With situs solitus, dextrocardia is associated with the normal orientation of great arteries. With situs inversus, the great vessels are usually inversely placed in the thoracic cavity. Furthermore, with situs ambiguous, the presence and origin of great vessels from ventricles are variable and usually accompanied by asplenia or polysplenia (4). Notably, dextrocardia with situs inversus occurs in approximately 2 per 10,000 live births. Dextrocardia solitus or situs ambiguous is considerably less common, occurring in 1 in 20,000 live births (5). Wolff-Parkinson-White (WPW) syndrome is an inborn abnormality with an additional electrical conduction pathway between the atria and the ventricle, which affects up to three in 1,000 people worldwide (6). Therefore, the occurrence of the two conditions is uncommon. These two congenital anomalies can occur alone, or in concurrence. Dextrocardia can be associated with other additional cardiac anomalies (7). However, no clear evidence of a significant correlation relationship between dextrocardia and atrioventricular nodal reentrant tachycardia or atrioventricular reentrant tachycardia was found. Likewise, WPW syndrome occurs as often in patients with dextrocardia as in the general populations. Klug D (8) reported that a patient with mirror-image dextrocardia and WPW Syndrome underwent catheter ablation successfully in 1994 for the first time. Experience of RFCA in patients with tachyarrhythmia and dextrocardia is still limited due to its infrequent incidence. This paper presented the case of a patient with both conditions in whom radiofrequency catheter ablation was performed successfully.

Situs inversus is a rare congenital heart condition that can be divided into situs inversus totalis and situs inversus with levocardia (9, 10). According to chest X-ray and color Doppler echocardiography results, the patient reported in our case is cardiac dextrocardia, accompanied by visceral inversion. The aortic arch and great vessels are all mirror-inverted. Thus, the heart is located in the right of the chest, the apex of the heart points to the right, the aortic arch in the right of the chest, and the inferior vena cava are left-sided structures. Catheter ablation is an effective and safe strategy with a high success rate for managing patients suffering from WPW syndrome and dextrocardia (11). A previous study involving nine cases of patients with supraventricular tachycardia and dextrocardia showed that the successful rate of catheter ablation was 100% (12). Therefore, for situs inversus without additional anatomy malformations, adjusting for projection angulation and catheter manipulation are the key points to achieving a successful ablation (13).

In this particular case, the main challenge of the procedure is locating the ablation target accurately. The key point to a successful ablation in patients with dextrocardia is understanding the heart’s anatomical structure nd the great vessels and identifying a strategy before conducting the operation. Relevant literature reported that the femoral vein is the most common ablation pathway in supraventricular tachycardia patients with dextrocardia when needed to RFCA (14, 15). In some cases, the superior vena cava is also the venous access to the heart (12). Fluoroscopy and 3D mapping during the procedure can help successfully ablate the accessory pathway in WPW syndrome. Besides, since mirror manipulation is required during the procedure in patients with supraventricular tachycardia and dextrocardia, catheter maneuvers are also crucial to a successful ablation.

## Conclusion

Wolff-Parkinson-White syndrome with dextrocardia has been reported previously with left free wall, right free wall, and anteroseptal accessory pathways (16). We report a unique case of a posteroseptal accessory pathway in a patient with dextrocardia and situs invertus. An exact anatomical evaluation is critical in such a case with dextrocardia. Therefore, EGM, chest-X ray, cardiac CT, cardiac color Doppler echocardiography, and 3D mapping can help guide the catheter to the ablation target, reduce the procedure duration, and decrease the risks of complications.

## Data availability statement

The original contributions presented in this study are included in the article/supplementary material, further inquiries can be directed to the corresponding author.

## Ethics statement

The studies involving human participants were reviewed and approved by the Research Ethics Committee of the Second Hospital of Hebei Medical University. The patients/participants provided their written informed consent to participate in this study. Written informed consent was obtained from the individual(s) for the publication of this case report and any potentially identifiable images or data included in this article.

## Author contributions

LZ: conceptualization, data collection and curation, and writing-original draft. RL and LB: methodology. DW: visualization. JZ: validation. XY: writing-review and editing. All authors contributed to the article and approved the submitted version.

## References

[1] Janson CM Millenson ME Okunowo O Dai D Christmyer Z Tan RB Incidence of life-threatening events in children with wolff-parkinson-white syndrome: analysis of a large claims database. *Heart Rhythm.* (2022) 19:642–7. 10.1016/j.hrthm.2021.12.009 34902591

[2] Kounis NG Zavras GM Papadaki PJ Soufras GD Kitrou MP Poulos EA. Pregnancy-induced increase of supraventricular arrhythmias in wolff-parkinson-white syndrome. *Clin Cardiol.* (1995) 18:137–40. 10.1002/clc.4960180306 7743683

[3] Xu J Jiang G Zhang L Chen Z Wang H Bai M Successful percutaneous left atrial appendage occlusion for atrial fibrillation in a patient with mirror-image dextrocardia: a case report. *BMC Cardiovasc Disord.* (2022) 22:20. 10.1186/s12872-021-02369-9 35090397 PMC8800237

[4] Al-Khadra AS. Images in cardiovascular medicine. Mirror-image dextrocardia with situs inversus. *Circulation.* (1995) 91:1602–3. 7867203 10.1161/01.cir.91.5.1602

[5] Offen S Jackson D Canniffe C Choudhary P Celermajer DS. Dextrocardia in adults with congenital heart disease. *Heart Lung Circ.* (2016) 25:352–7. 26541676 10.1016/j.hlc.2015.09.003

[6] Elitok A Aksan G Sonsöz MR Tezcan M Çevrim Ö. The coexistence of wolff-parkinson-white syndrome (WPW) and atrioventricular nodal reentrant tachycardia (AVNRT). *Turk J Emerg Med.* (2018) 18:131–3. 10.1016/j.tjem.2017.12.002 30191196 PMC6107921

[7] Shi H Sohn S Wang S Park S Lee S Kim SY A case of multiple cardiovascular and tracheal anomalies presented with wolff-parkinson-white syndrome in a middleaged adult. *J Korean Med Sci.* (2017) 32:2069–72. 10.3346/jkms.2017.32.12.2069 29115093 PMC5680510

[8] Klug D Dubuc M Ferracci A Nadeau R. Radiofrequency catheter ablation of an accessory pathway in a young man with dextroversion. *Pacing Clin Electrophysiol.* (1994) 17:981–5. 10.1111/j.1540-8159.1994.tb01443.x 7517535

[9] Karki S Khadka N Kashyap B Sharma S Rijal S Basnet A. Incidental finding of dextrocardia with situs inversus and absent left kidney: a case report. *JNMA J Nepal Med Assoc.* (2022) 60:196–9. 10.31729/jnma.6825 35210643 PMC9200005

[10] Goo HW Park IS Ko JK Kim YH Seo DM Yun TJ CT of congenital heart disease: normal anatomy and typical pathologic conditions. *Radiographics.* (2003) 23:S147–65. 14557509 10.1148/rg.23si035501

[11] Zhang Y Sun L Luo F Li J Sun Y Chen Y Result and technique consideration of radiofrequency catheter ablation of tachycardia in patients with dextrocardia. *Pacing Clin Electrophysiol.* (2022) 45:340–7. 10.1111/pace.14452 35044698

[12] Zhou GB Ma J Zhang JL Guo XG Yang JD Liu SW Catheter ablation of supraventricular tachycardia in patients with dextrocardia and situs inversus. *J Cardiovasc Electrophysiol.* (2019) 30:557–64. 30661266 10.1111/jce.13847

[13] Ulus T Dural M Şener E Al A Mert KU Görenek B. Atrial fibrillation and atrial flutter ablation using mirror image in a patient with dextrocardia with situs inversus. *Anatol J Cardiol.* (2020) 24:282–4. 10.14744/AnatolJCardiol.2020.20766 33001046 PMC7585963

[14] Povoski SP Khabiri H. Persistent left superior vena cava: review of the literature, clinical implications, and relevance of alterations in thoracic central venous anatomy as pertaining to the general principles of central venous access device placement and venography in cancer patients. *World J Surg Oncol.* (2011) 9:173. 10.1186/1477-7819-9-173 22204758 PMC3266648

[15] Zheng Z Zeng Z Zhou Y Li C Zhang W. Radiofrequency catheter ablation in a patient with dextrocardia, persistent left superior vena cava, and atrioventricular nodal reentrant tachycardia: a case report. *Medicine.* (2020) 99:e22086. 10.1097/MD.0000000000022086 32899085 PMC7478451

[16] Chin A Phiri T. An anteroseptal accessory pathway in a patient with dextrocardia and situs inversus. *J Cardiovasc Electrophysiol.* (2015) 26:692–3. 10.1111/jce.12616 25588496

